# P169: Is patient participation useful to improve staff hand hygiene compliance in a geriatric hospital?

**DOI:** 10.1186/2047-2994-2-S1-P169

**Published:** 2013-06-20

**Authors:** V Sauvan, Y Registe Rameau, L Pagani, D Pittet

**Affiliations:** 1 Infection Control Program, Geneva University Hospitals, Geneva, Switzerland; 2 DMIRG, Geneva University Hospitals, Geneva, Switzerland

## Objectives

Patient participation to enhance compliance with hand hygiene practices may improve the global strategy. We investigated the ability of geriatric patients to be involved in such a strategy.

## Methods

We conducted a survey on the impact of feedback and patient participation on hand hygiene compliance at our institution in 2012 and included 5 of 16 wards at the geriatric hospital. Patients were randomly recruited by an infection control nurse in 2 of the 5 wards to investigate the potential for patients to be actively engaged in hand hygiene practices. Inclusion criteria were: a cognitive capability of >19 as measured by a Mini Mental State examination and a stable health condition. At day 0, patients were trained in hand hygiene indications that should be carried out by staff and by themselves. Patients were given a training brochure and a bottle of alcohol-based handrub, together with a questionnaire about their willingness to either carry out hand hygiene or remind staff of the procedure, if not accomplished. At day 7, included patients underwent an interview to assess their active participation.

## Results

91 patients were screened; 39 fulfilled inclusion criteria and 20 accepted to participate. At day 0, participants declared their willingness to comply with between 35% to 95% of the social recognized and trained indications related to personalhand hygiene. Four of 20 patients agreed to remind staff of hand hygiene indications if necessary. At day 7, 7 of 16 patients still hospitalized recalled their engagement; 11 confirmed the use of alcohol-based handrub and none had reminded staff.

## Conclusion

Patient participation in hand hygiene practices remains low among the elderly. Elderly patients can be taught correct self-use of alcohol-based handrub, but reminding staff to comply remains quite difficult. Training the elderly with accessible and targeted information might also positively impact on staff perception and relieve workload.

## Disclosure of interest

None declared

